# Emergency Radiology in the First 24 h of Two Major Earthquakes on the Same Day and Radiologic Evaluation of Trauma Cases

**DOI:** 10.3390/tomography10080099

**Published:** 2024-08-22

**Authors:** Mehtap Ilgar, Nurullah Dağ

**Affiliations:** 1 Department of Radiology, Ankara Etlik City Hospital, Ankara 06170, Turkey; 2 Department of Radiology, Faculty of Medicine, İnönü University, Malatya 44000, Turkey; drndag@icloud.com

**Keywords:** earthquake, trauma, emergency radiology

## Abstract

Background: On 6 February 2023, two major earthquakes occurred in Turkey on the same day. More than 50,000 people died, and more than 100,000 people were injured in these earthquakes. The aim of this study is to contribute to disaster management plans by evaluating the functioning of a radiology department and the imaging examinations performed after this disaster. Methods: The functioning of the radiology clinic at Malatya Training and Research Hospital in the first 24 h after the earthquake was evaluated. The images of 596 patients who were admitted to Malatya Training and Research Hospital for earthquake-related trauma between 6 February 2023, at 4:17 a.m. and 7 February 2023, at 4:17 a.m., and who underwent radiography and computed tomography (CT) were retrospectively reviewed. Results: The mean age of the patients was 37.3 ± 20.1 years. A total of 313 (52.5%) patients were male. The most frequently performed imaging test was a CT scan. In total, 437 (73.3%) of 596 patients underwent a CT scan. At least one body part was affected in 160 patients (26.8%). The most commonly affected regions were the thorax, vertebrae, and extremities. Thoracic findings were observed in 52 patients (32.5%), vertebral findings in 52 patients (32.5%), and extremity findings in 46 patients (28.7%). Fractures were the most common finding in our study. Of the 160 patients with pathologic findings, 139 (86.9%) had evidence of fractures. Conclusions: The role of radiology in disasters is important. When disaster preparedness plans are made, radiology departments should be actively involved in these plans. This will ensure the quick and efficient functioning of radiology departments.

## 1. Introduction

On 6 February 2023, two earthquakes of magnitude 7.7 and 7.6 with epicenters in Pazarcık (Kahramanmaraş) and Elbistan (Kahramanmaraş) occurred at 04:17 and 13:24, respectively [1]. More than 50,000 people lost their lives, and more than 100,000 people were injured in these earthquakes [2]. Malatya province was one of the places affected by these two major earthquakes and was declared a disaster area. The hospital where this study was performed is the largest trauma center in Malatya.

Earthquakes are serious and unpredictable natural disasters. Various conditions, such as fractures, organ and soft tissue injuries, cardiovascular diseases, pulmonary diseases, and infectious diseases, can occur following earthquakes [3].

Digital radiography, ultrasound (US), computed tomography (CT), and magnetic resonance imaging (MRI) are essential imaging modalities for the rapid and reliable assessment of earthquake-related injuries [3]. However, MRI should not be utilized immediately following an earthquake due to the risk of quenching. Quenching involves the rapid release of liquid cryogen that maintains the MRI magnet in a superconducting state. If the gas leaks into an enclosed space rather than venting outside after an earthquake or other disaster, there is a risk of suffocation and freezing [4].

Bone fractures are the most common type of earthquake-related injury, comprising approximately half of all such injuries [5]. CT is the preferred imaging modality for patients with major or multisystem trauma. Studies have shown that patients undergoing standardized whole-body CT have significantly better outcomes [6,7]. Ultrasonography is the most useful and accessible imaging modality, both during the disaster and within the subsequent six hours. This is primarily due to the significant energy requirements of conventional X-ray and CT scanners [8].

Radiology departments play a complementary role in responding to mass casualty events. They serve to clarify diagnoses, prevent an incomplete or excessive triage of patients, and provide timely communication of imaging results to the clinical teams providing primary care [9]. 

The aim of this study is to contribute to disaster preparedness plans by evaluating the functioning of a specific radiology clinic and the imaging studies performed after two major earthquakes on the same day.

## 2. Materials and Methods

The functioning of the radiology department of Malatya Training and Research Hospital in the first 24 h after the earthquake was evaluated using hospital records, information obtained from radiology staff through personal interviews, and personal observations by the authors of the article. 

Between 6 February 2023 at 4:17 a.m. and 7 February 2023 at 4:17 a.m., the radiology records of all patients admitted to Malatya Training and Research Hospital for earthquake-related trauma and who underwent radiography and CT scans were retrospectively reviewed. The X-rays and CT scans of all patients were retrospectively reviewed by two radiologists from the hospital’s archive imaging system, and a consensus decision was reached. The CT images were acquired using two 128-slice GE Healthcare CT scanners (Madison, WI, USA) and one 16-slice Toshiba CT scanner (Tokyo, Japan). Radiographic images were obtained using equipment from DRGEM GXR (Seoul, Republic of Korea), Drs SYTEC DR, Sedecal (Madrid, Spain), and Shimadzu (Kyoto, Japan). Two patients were excluded from this study because of artifacts in their CT images. A total of 596 patients were included in this study. The affected body parts were divided into 7 regions, including thorax, vertebrae, extremities, pelvis, head, abdomen, and other (paranasal sinus, nasal, orbit, maxilla, mandible, and zygoma). Soft tissue findings were not included in this study. US findings were not included in our study. This was because the evaluations conducted with portable devices in the emergency room were briefly recorded on paper. As this information was not transferred to the hospital data system, the data were not accessible.

### 2.1. Statistical Analysis

Statistical analysis was performed with SPSS version 22 (SPSS Inc., Chicago, IL, USA). Means and standard deviations were calculated for continuous variables and counts and percentages were calculated for categorical data. Patients were divided into two groups: 0–17 years and ≥18 years. A Chi-squared test was performed to compare age groups based on affected areas. A *p* value of <0.05 was considered to be statistically significant. 

### 2.2. Ethical Approval

This study was approved by the Non-Interventional Clinical Research Ethics Committee of Malatya Turgut Özal University (approval number E-30785963-020-154286 on 11 April 2023).

## 3. Results

The first earthquake occurred at 04:17. At that time, only on-call radiologists and on-call radiology technicians were in the radiology clinic. Within half an hour, the patient load increased, and all available radiology personnel were called to emergency duty. Meanwhile, despite the power outage, the generators were on, and the imaging equipment was working. Five X-ray units, two 128-detector CT units, and one 16-detector CT unit were available. 

Routine radiology services were suspended. A shift work schedule was established for the staff. Imaging equipment was located on the ground floor, where the emergency department was located. Ultrasound equipment in the radiology department was moved to the emergency department. Radiologists worked with other specialists in the emergency department. Ultrasound reports were handwritten on paper or verbally communicated to the physician face-to-face. The CT reports were also communicated verbally to the clinician face-to-face.

Patients ranged in age from 0 to 100 years, with a mean age of 37.3 ± 20.1 years. In total, 313 (52.5%) were male and 283 (47.5%) were female. A total of 499 (83.7%) patients were 18 years of age or older. The demographic characteristics of the patients are shown in Table 1.

A CT scan was the most commonly performed examination. A total of 437 (73.3%) out of 596 patients underwent a CT scan. The distribution of radiographic and CT examinations is shown in Table 2. The majority of CT scans were whole-body CT scans performed without contrast. Contrast was used only when clinically indicated.

**Figure 1:**
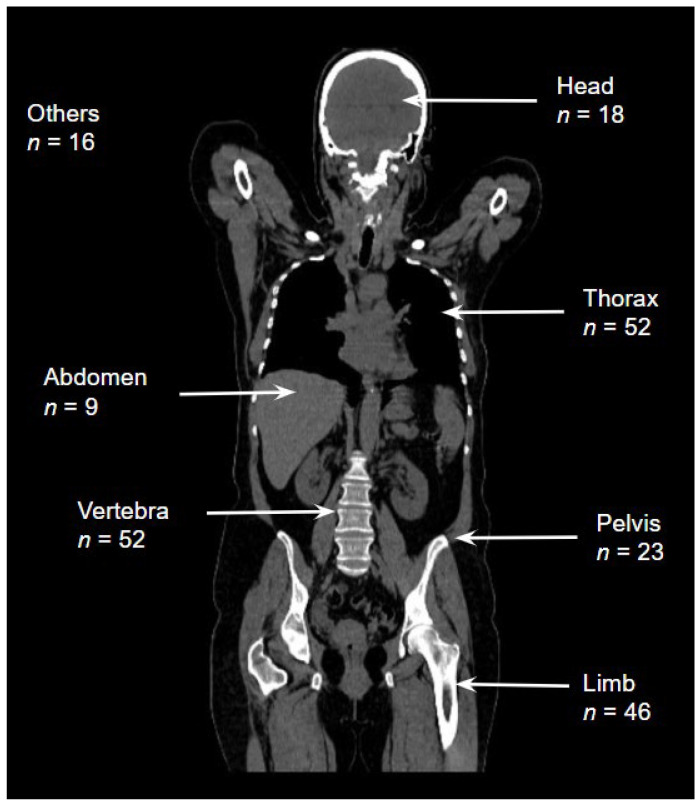
The number of patients affected by region.

The most commonly affected regions were the thorax, vertebrae, and extremities. The number of patients affected by region and age group is shown in Table 3 and Figure 1.

No region was affected in 436 patients (73.2%). In 115 (19.3%) patients, one site was affected; in 35 (5.9%) patients, two sites were affected; in 9 (1.5%) patients, three sites were affected; and in 1 (0.2%) patients, four sites were affected. 

**Figure 2:**
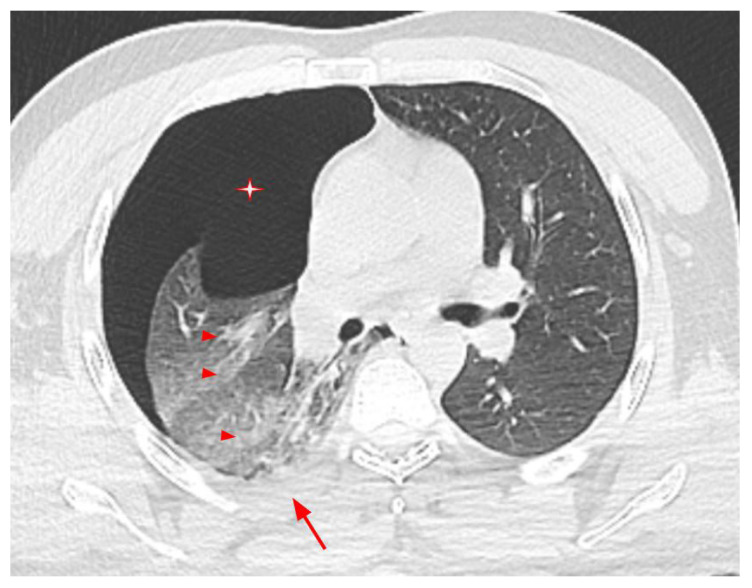
A 32-year-old male patient with pneumothorax (asterisk), hemothorax (long arrow), and parenchymal contusion areas (arrowheads).

**Figure 3:**
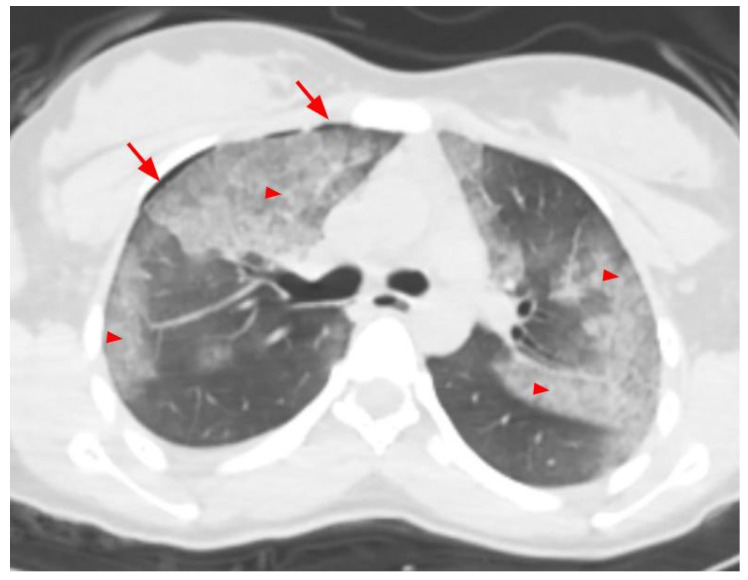
A 22-year-old female patient with areas of contusion in bilateral lung parenchyma (arrowheads) and pneumothorax on the right (arrows).

Contusions were most common in patients with thoracic findings. Contusions were seen in 35 (67.3%) patients, rib fractures in 21 (40.4%), pneumothorax in 20 (38.5%), hemothorax in 18 (34.6%), and laceration in 3 patients (5.8%). In 26 (50%) patients, multiple thoracic findings were seen together. A CT image of a patient with thorax findings is presented in Figure 2 and Figure 3.

**Figure 4:**
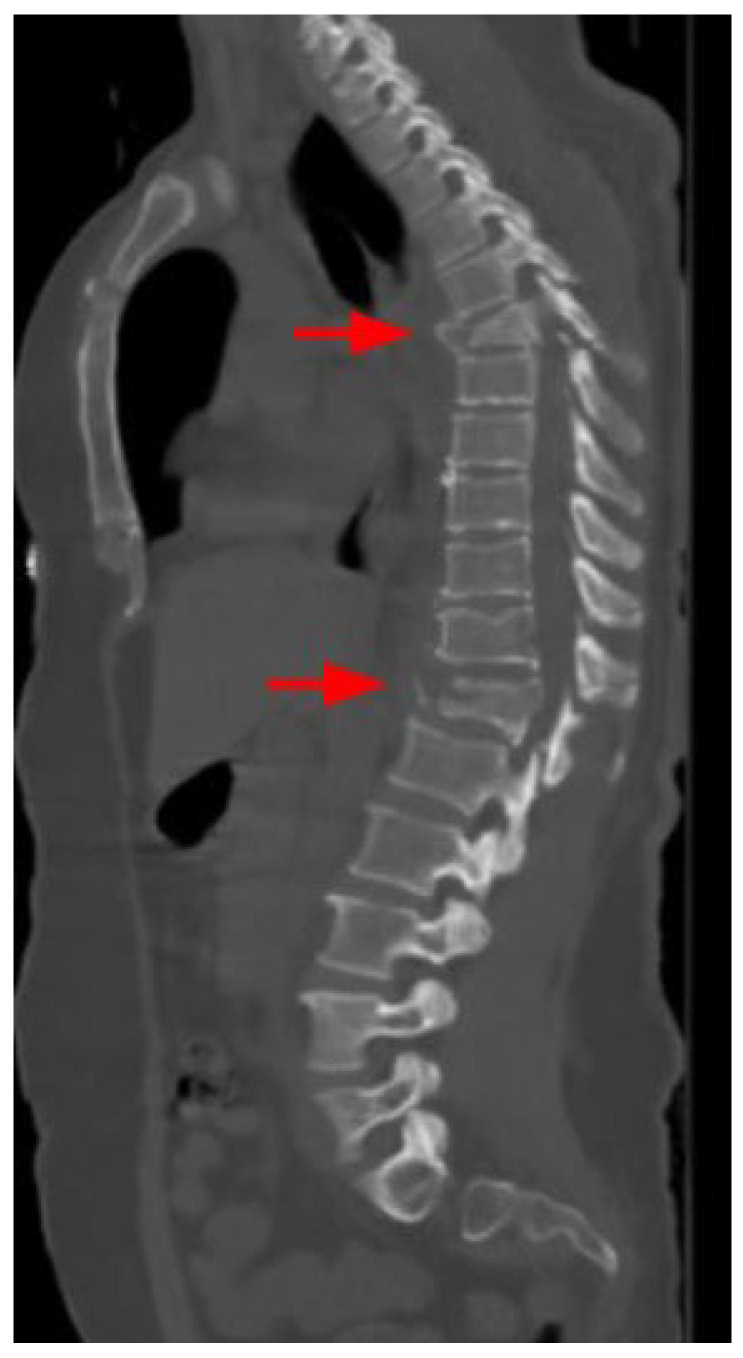
A 43-year-old female patient with fracture lines (red arrows) in T6 and 12 vertebral bodies.

Among the patients with vertebral fractures, 35 (67.3%) had lumbar fractures, and 18 (34.6%) had thoracic fractures. A CT image of a patient with a vertebral fracture is presented in Figure 4.

**Figure 5:**
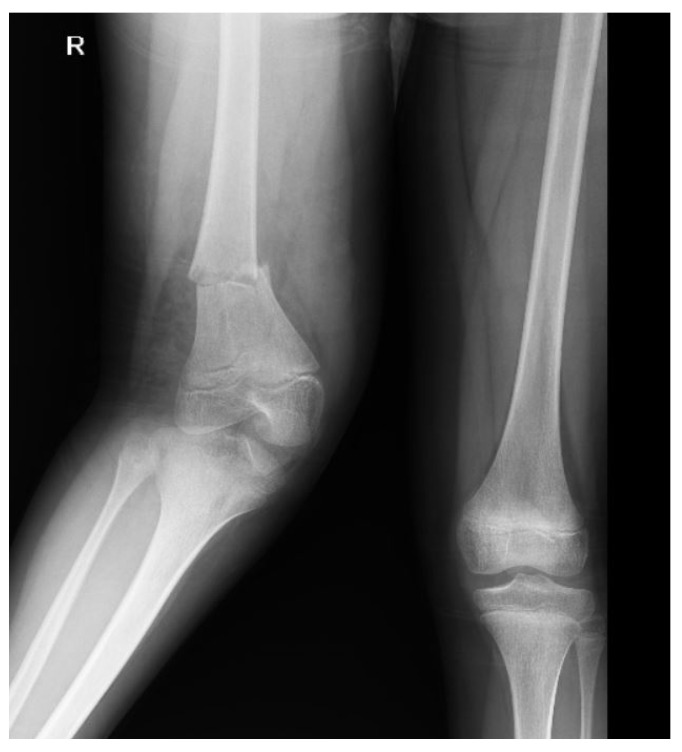
A 9-year-old male patient with a right femur distal diaphyseal fracture.

The most common extremity findings were fibula fractures in 11 (23.9%) patients and femur fractures in 10 (21.7%) patients. Figure 5 shows a radiography image of a patient with an extremity fracture.

**Figure 6:**
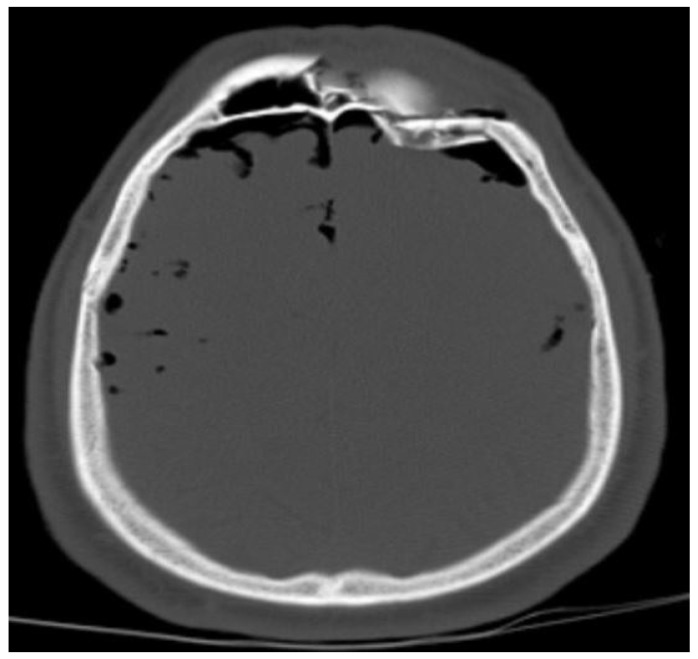
A 59-year-old male patient with a depressed fracture of the frontal bone and pneumocephaly.

There was a difference in the affected areas between children and adult patients only in the head region. The rate of pathology in the head region was significantly higher in pediatric patients than in adult patients (*p* = 0.027). Figure 6 shows the CT image of a patient with head findings.

## 4. Discussion

Many factors are needed for the radiology department to function fully, including personnel, infrastructure, equipment, supplies, and information technology. The first earthquake occurred at 04:17 local time, and at that time, there was one radiologist and seven radiology technicians on duty at the hospital. It was soon realized that additional staff were needed, and other radiology staff were called into service. On the day of the earthquake, it was cold in Malatya, and the roads were icy. Traffic was very heavy as everyone tried to get out of the city center. Although these factors made access to the hospital difficult, a sufficient number of radiology staff were able to reach the hospital on their own. In the aftermath of a major disaster, some personnel may be unable to work due to personal or family injuries or may face significant barriers to travel [10]. In the event of road closures, prearranged off-site parking for personnel or prearranged transportation from emergency services can be provided [10]. Hospital staff may be affected by disasters, and their absence or unpreparedness may affect the continuity of services in emergency situations caused by disasters. Therefore, they are expected to be aware of their role in the implementation of disaster plans [11].

In recent years, imaging has been increasingly used in the evaluation of critically ill patients. Excessive triage can seriously affect resource availability, and inadequate triage can seriously affect mortality. Therefore, imaging is important for increasing the accuracy of prioritization [12]. Another role of radiology is the rapid, appropriate, and accurate communication of imaging findings [13]. 

The location of radiology units is important in disaster situations and may vary according to regional risks [14]. In our hospital, X-ray and CT machines were located on the ground floor near the emergency department. This location was an important advantage because some of the elevators were damaged in the earthquake and did not work. If it had been necessary to move to another floor, the elevators would have been backed up, and it would have been difficult to reach the equipment. In addition, there were continuous large aftershocks, and it was very risky to use the elevators while the aftershocks continued. Since the radiology department was on the ground floor, there was no need for an elevator. The CT and X-ray machines were close to the emergency unit, which allowed for easy and quick access.

CT was the most requested test, and the number of patients needing CT units increased. The priority was determined according to the clinical situation. However, some patients and their relatives who thought that their own conditions should be prioritized caused verbal arguments in front of the CT unit. Although there was no physical attack in our hospital, it should be considered that security problems may occur in hospitals during disasters. Measures to prevent security problems should be included in disaster plans. 

The second earthquake occurred at 1:24 p.m. local time. No one expected it. Since the first earthquake occurred outside of working hours, the radiology staff who were with their families during the first earthquake were away from their families during the second earthquake and worried about them as well. Meanwhile, the aftershocks continued, and the anxiety and fear of the building collapsing increased. The second earthquake shows that worst-case scenarios should always be considered when planning for disasters. When preparing for these scenarios, one of the factors to consider is that the personnel who will be the first responders are also disaster victims. Even if these personnel are physically healthy, the fact that they will be emotionally affected should not be forgotten. 

Ultrasonography was the most useful and accessible imaging modality both at the time of the disaster and for the next six hours. The main reason for this is the energy requirements of imaging modalities [4]. Conventional X-ray and CT scanners require a significant amount of electrical power when activated. Therefore, hospitals in disaster areas may face the risk of a complete power failure when activating these radiological diagnostic devices. Hospitals that do not have an emergency power plan in place will likely be unable to activate these devices [8]. Portable US can facilitate focusing. It can be used for the sonographic assessment of trauma for surgical triage, hemothorax/pneumothorax, solid organ injury, pregnancy, vascular injury assessment, and pediatric head assessments [15]. Focused assessments with sonography for trauma (FAST) ultrasound have been established to rapidly screen for life-threatening injuries that inevitably require surgery without the need for further diagnosis [16]. Since the majority of USs performed within the first 24 h at our hospital are not recorded in the hospital information system, there is no information regarding the number of patients who underwent US or the associated findings. USs were performed by radiologists in designated areas. The number of computers in these areas was limited and primarily used for other essential tasks. The hospital’s computers were fixed units, and there were no suitable computers nearby where radiologists could access the hospital information system to enter reports. Consequently, US reports were manually recorded on paper for a period. The paper reports included the patient’s identity information, the time of the US, the location of the examination, and the findings. The study by Erdemir et al. also noted that in necessary situations, reports can be written on paper, and this method may be used [17]. Additionally, some patients’ reports were delivered to clinicians face-to-face. Face-to-face delivery was particularly beneficial for ‘red zone’ patients, as it allowed clinicians to receive results immediately.

CT is the imaging modality of choice for patients with major or multiple-system trauma. Patients undergoing standardized whole-body CT have been shown to have significantly better outcomes [6,7]. The use of pan–scan has been reported to be an independent predictor of survival [18].

CT is the most reliable and fastest examination with which to analyze an emergency patient [19]. However, the large number of casualties in a mass casualty event can result in a wait time for both the acquisition and visualization of the data sets. To prepare for multiple traumas, hospitals should have a CT scanner in the emergency department, ideally with direct access from the resuscitation area [20]. Mobile CT is an effective imaging modality that can be used in both standard hospitals and field hospitals. Mobile CT can be deployed in two different ways: portable units within the hospital and vehicle-mounted units for field use. Portable CT offers significant advantages in areas where patient transport is difficult and risky, such as trauma centers, operating rooms, and intensive care units [21]. Vehicle-mounted mobile CT technology provides a rapidly deployable and operable CT unit in extraordinary situations, such as earthquakes, wars, and pandemics [22]. In emergency departments, portable CT units are preferred for high-energy multi-trauma cases, whereas vehicle-mounted mobile CT may be more appropriate in situations where structural damage or technical failures occur in the hospital building. Reports of radiological experience following the Christchurch earthquake indicate that power outages or problems with backup power sources delayed initial CT scans by approximately 5 h [23]. In this context, both methods of mobile CT can be effective and beneficial in extraordinary circumstances such as earthquakes. 

Imaging studies should be reported promptly, immediately after imaging, to investigate life-threatening injuries that may require urgent intervention, such as tension pneumothorax or active hemorrhage, or conditions, including diffuse intracranial hemorrhage, which may significantly alter the immediate clinical course or prognosis of the patient [24]. 

CT equipment was operational in our hospital, and CT examinations were performed on 437 patients during the first 24 h. The reports of these examinations were personally communicated by the radiologist to the attending physician. 

The most common finding in earthquake victims is a fracture [5,25]. The most common finding in our study was also fractures. Of the 160 patients with pathological findings, 139 (86.9%) had evidence of fractures. Fractures were observed in a single bone in 84 (60.4%) patients and in more than one bone in 55 (39.6%) patients. The tibia, fibula, and femur are the bones with the most frequent fractures [26]. Similarly, in our study, the most commonly fractured bones were the fibula, femur, and tibia.

While the most common findings in the literature are in the extremities, in our study, thoracic and vertebral findings were seen at a higher rate than the extremities. This may be due to the high utilization of CT scans at 73.3%. In our study, contusion was most commonly seen in patients with thoracic findings. In total, 67.3% of patients with thoracic findings had contusions. This high rate of contusion may also be explained by the use of CT. Earthquake-related lung parenchymal findings are often associated with rib fractures [27]. In our study, 21 (40.4%) patients with chest findings had rib fractures.

Spinal fractures need to be recognized urgently because they may cause serious morbidity and require surgical intervention [28]. The thoracolumbar junction is the most common site of vertebral injury in earthquakes [29,30]. Consistent with the literature, the most common fracture in our study was at the thoracolumbar junction. Today, artificial intelligence applications are increasingly being integrated into the field of medical imaging. Artificial intelligence can be a valuable tool for radiologists, especially in challenging and stressful environments. In emergency situations, AI can assist in worklist prioritization, image interpretation, and structured reporting [31]. It has been demonstrated that vertebral body fractures can also be detected using artificial intelligence. One study showed that vertebral body fractures were automatically identified using a developed neural network, with a high diagnostic performance to assess fracture stability [32].

Head injuries account for 1.8–25.7% of injuries in post-earthquake trauma patients and usually require urgent intervention [33]. In our study, this rate was 11.3%. The three most common findings were fracture, pneumocephaly, and subdural hemorrhage.

### Limitations

Our study is single-center and includes patients who presented at the hospital in the first 24 h after the earthquake. The affected areas are reported, but injury severity is not specified. Information on patient prognosis is not included. Despite all these limitations, we believe that our study can contribute to radiology plans for disaster management preparedness.

## 5. Conclusions

In our study, the most common findings were fractures, with the thorax, vertebrae, and extremities being the most frequently affected body regions. This major disaster has highlighted the crucial role of radiology in managing patients during emergency situations. Radiology departments should recognize their critical importance and be adequately prepared for such scenarios. When developing disaster preparedness plans, radiology departments should be actively involved to ensure their prompt and effective operation.

## Figures and Tables

**Table 1:** Demographic characteristics of patients.

	*n* (%)
Male	313 (52.5)
Female	283 (47.5)
0–17 age	97 (16.3)
≥18 age	499 (83.7)
	596 (100)

**Table 2:** Number and percentage of patients who received radiographs and CT scans.

Image Modality	*n* (%)
CT	363 (60.9)
Radiography	159 (26.7)
CT and Radiography	74 (12.4)
	596 (100)

**Table 3:** Situations of concern by region and age group.

	0–17 Age (*n* = 19)		≥18 Age (*n* = 141)		All Ages (*n* = 160)		*p*
	Finding + *n* (%)	Finding − *n* (%)	Finding + *n* (%)	Finding − *n* (%)	Finding + *n* (%)	Finding − *n* (%)	
Thorax	7 (36.8)	12 (63.2)	45 (31.9)	96 (68.1)	52 (32.5)	108 (67.5)	0.667
Vertebra	3 (15.8)	16 (84.2)	49 (34.8)	92 (65.2)	52 (32.5)	108 (67.5)	0.098
Limb	5 (26.3)	14 (73.7)	41 (29.1)	100 (70.9)	46 (28.7)	114 (71.3)	0.803
Pelvis	1 (5.3)	18 (94.7)	22 (15.6)	119 (84.4)	23 (14.4)	137 (85.6)	0.228
Head	5 (26.3)	14 (73.7)	13 (9.2)	128 (90.8)	18 (11.3)	142 (88.7)	0.027
Abdomen	1 (5.3)	18 (94.7)	8 (5.7)	133 (94.3)	9 (5.6)	151 (94.4)	0.942
Others	1 (5.3)	18 (94.7)	15 (10.6)	126 (89.4)	16 (10.0)	144 (90.0)	0.463

## Data Availability

Data are contained within the article.
